# Masseter Muscle Metastasis of Renal Cell Carcinoma: A Case Report and Literature Review

**DOI:** 10.3389/fonc.2022.830195

**Published:** 2022-02-02

**Authors:** Fei Qin, Xiaofei Zhang, Jie Zhang, Shuaihong Liu, Zijie Wang, Fei Xie, Mingxin Zhang, Tianwei Zhang, Shuangyi Wang, Wei Jiao

**Affiliations:** ^1^ Department of Urology, The Affiliated Hospital of Qingdao University, Qingdao, China; ^2^ Department of Education and Training, The Affiliated Hospital of Qingdao University, Qingdao, China; ^3^ Department of Endocrinology and Metabolism, The Affiliated Hospital of Qingdao University, Qingdao, China; ^4^ Department of Stomatology, The Affiliated Hospital of Qingdao University, Qingdao, China

**Keywords:** masseter muscle, muscle metastasis, renal cell carcinoma, case report, literature review

## Abstract

**Background:**

Patients with renal cell carcinoma are often troubled by metastases, but masseter muscle metastases are particularly rare.

**Case Presentation:**

We reported a 76-year-old male who did not show any recurrence and metastasis after the nephrectomy until 5 years later. The metastatic mass was found with the protrusion of masseter muscle area. Computed tomography and ultrasonography indicated a hypervascular mass, and pathology confirmed the masseter muscle metastasis of renal cell carcinoma. Complete metastasectomy was performed with the preserval of facial function and appearance. No local recurrence or distant metastasis was found in follow-up.

**Conclusion:**

Our report indicates masseter muscle can be a metastatic site of renal cell carcinoma, regardless of its rarity. Long-term comprehensive surveillance is needed for patients with renal cell carcinoma. Muscle metastases can disguise as benign mass, while multiple imaging and pathology are important in identifying their sources. If possible, complete metastasectomy with function retention is recommended for masseter muscle metastases.

## Introduction

Renal cell carcinoma (RCC) is a common malignant tumor of the urinary system, with the highest mortality in urologic tumors (1). It is known that metastasis is an important factor contributing to death. About 16% of patients with RCC had distant metastases at the time of discovery (2), and about a quarter of patients with localized RCC eventually had distant metastases after the nephrectomy (3). Lungs, bones, lymph nodes, liver, adrenal glands, and brain were common metastatic sites (4), while skeletal muscles were rare sites, accounting for 0.4% in all metastatic sites (5). According to bodies literature, skeletal muscle metastases involved lower limbs (37.2%), upper limbs (25.6%), trunk (20.9%), and neck and head (16.3%), while muscle metastases in head and neck were the least (6).

We reported a case of masseter muscle metastasis five years after the nephrectomy. We reviewed relevant bodies of literature, to collect clinical characteristics and provide reference for the diagnosis and treatment of those patients.

## Case Presentation

**Figure 1:**
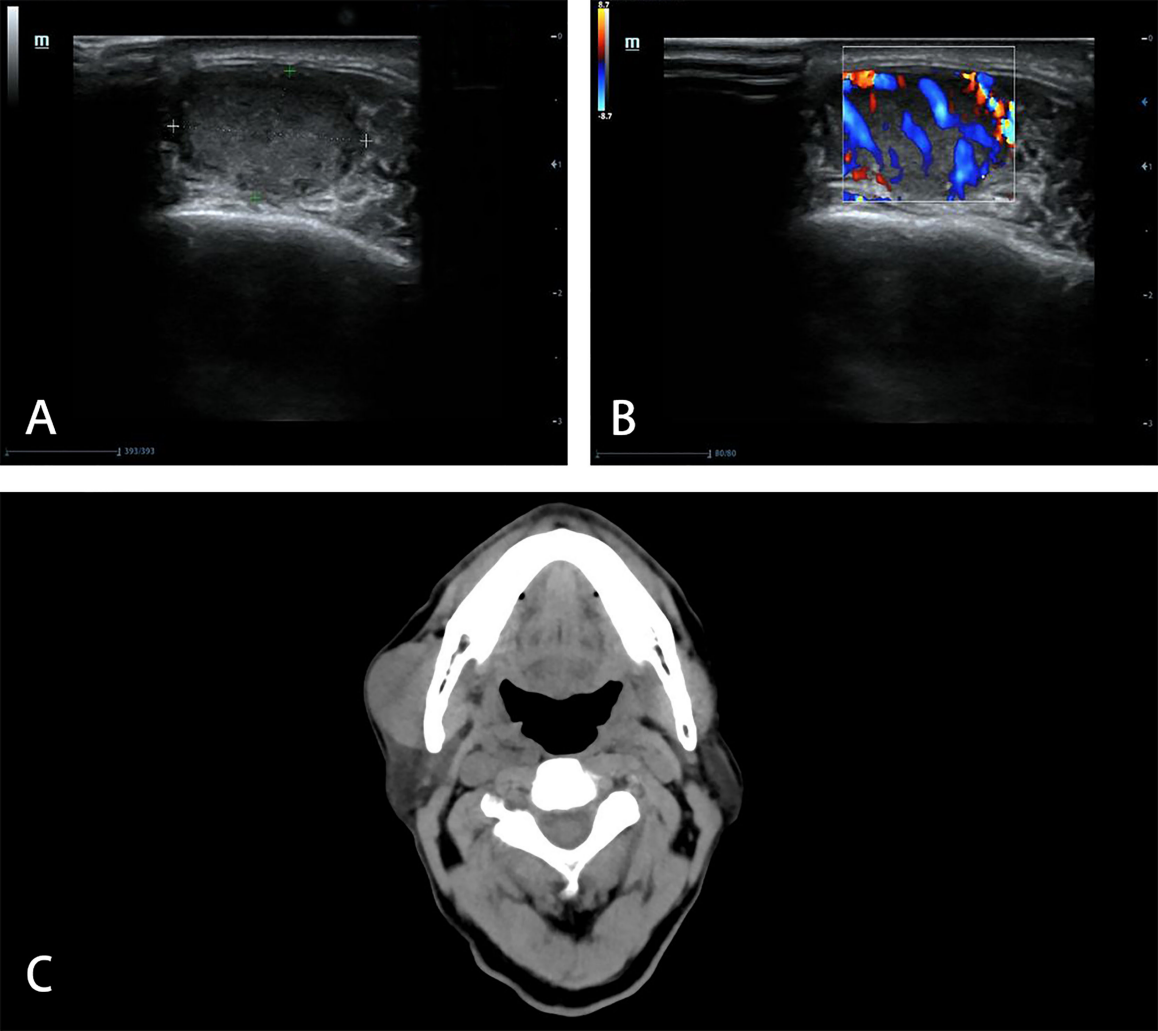
Imaging manifestations of masseter muscle metastasis in US and CT. US shows a hypoechoic **(A)**, hypervascular **(B)** mass in the muscle layer of the masseter area in 2019. CT shows unclear boundary between the mass and right masseter muscle in 2021 **(C)**.

A 76-year-old male was hospitalized in our hospital due to asthma in July 2019 and found a mass in the right masseter area without symptoms. Ultrasonography (US) showed a mass of 17 mm × 17 mm × 10 mm which could be detected in the muscle layer of the masseter area, with hypoechogenicity, clear boundary and high vascularity (**Figure 1**). A diagnosis of “fibroma” was made. The patient chose close follow-up rather than any treatment, because of concomitant asthma and painless mass.

The mass increased gradually, so he went to our hospital in June 2021. In the area of masseter muscle, a round protrusion with a diameter of 3 cm was touched and local skin was complete. It was a little tough in texture, with no tenderness and poor mobility. Computed tomography (CT) demonstrated that the solitary mass of 38.4 mm × 22.3 mm existed at the surface of masseter muscle and the boundary with the masseter muscle was unclear (**Figure 1**). There was no other mass found in CT.

The patient had a history of laparoscopic radical nephrectomy in September 2014. The tumor was located at the pole of the kidney. There was no invasion of vascular system, lymphatic system, broken end of ureter and renal capsule. The post-operative pathology demonstrated clear-cell RCC (ccRCC, T1N0M0, Furhman grade 2) of the left kidney. It is possible that the mass is a metastatic lesion from RCC. Other history included asthma with stable condition. The patient and his family had no history of tumor.

**Figure 2:**
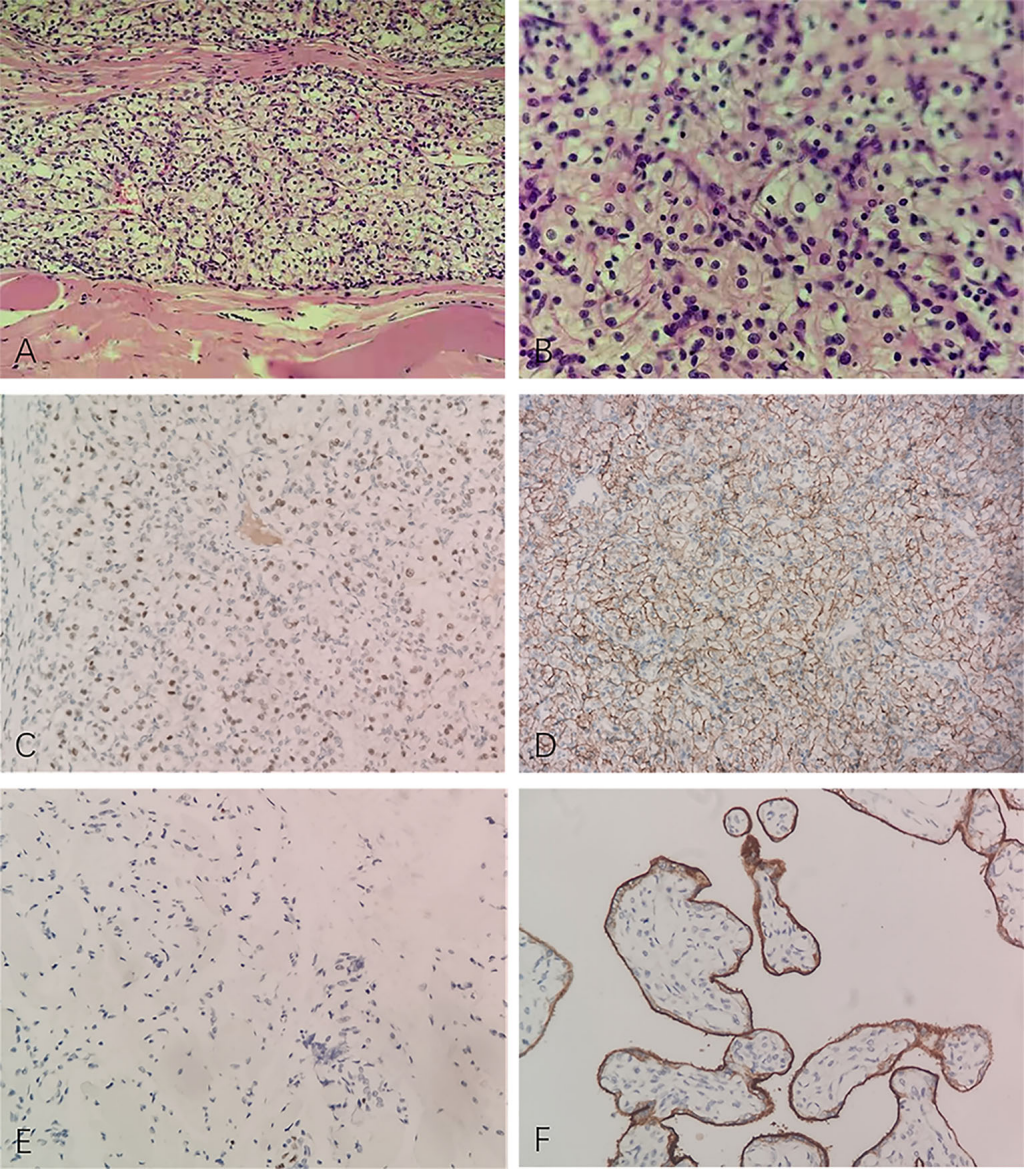
Pathological manifestations of masseter muscle metastasis. Hematoxylin–eosin staining (×200): tumor tissue infiltrates in striated muscle tissue, with rich blood sinuses **(A)**. Hematoxylin–eosin staining (×400): tumor cells share the morphology of clear cytoplasm **(B)**. Immunohistochemistry: Pax-8 **(C)**, CA IX **(D)**, Ki-67 (5%) **(E)** and PD-L1 (22c3, CPS ≈ 1) **(F)** are positive. The figure was obtained in 2021.

The patient asked to remove the mass. Then the patient underwent a surgery in June 2021, for diagnosis and treatment at the same time. The mass was found at the surface of the masseter muscle. The mass and part of the masseter muscle were removed. The superficial lobe of parotid gland was also removed due to partial involvement. The mass was gray and yellow in section, and about 30 mm × 30 mm × 25 mm in size. Frozen sections were positive for malignancy and negative for surgical margin. Post-operative pathology showed tumor cells had same morphology of clear cytoplasm, and tumor tissue infiltrated in striated muscle tissue, with rich blood sinuses. Immunohistochemistry revealed positive reactivity to Pax-8, CD10, CA IX, Ki-67 (5%), PD-L1 (22c3, CPS ≈ 1), and negative reactivity to CK7, CK19, p63, calponin, CD117, S100 (**Figure 2**). Combining morphology, immunohistochemistry and previous medical history, we made the diagnosis of masseter muscle metastasis of RCC.

There was no local recurrence and distant metastasis in follow-up positron emission tomography/computed tomography (PETCT) one month later. The patient did not complain about any abnormality of facial function or appearance.

## Discussion

The biological behavior of RCC is difficult to predict. Metastases often exist at the time of initial diagnosis or following surveillance. However, muscle metastases of RCC are very rare, which is consistent with high resistance of muscle to cancer. We reported a case of masseter muscle metastasis of RCC. According to what we had known, there were only 5 similar cases, which were not collected and overviewed before. We gathered their clinical characteristics in **Table 1**. Because of limited reports of masseter muscle metastases, cases with skeletal muscle metastases of RCC were searched and reviewed in PubMed and related references (**Supplementary Figure 1**), especially focusing on their diagnoses and treatments. The key words were “muscle”, “renal cell carcinoma” and “metastasis/metastases”, with the limit to English articles and the deadline of August 31, 2021. We collected more clinical characteristics and listed them in (**Supplementary Table 1**).

**Table 1:** Clinical characteristics of cases with masseter muscle metastasis of RCC.

Ref.	Age (years)/Gender	Interval^*^ (months)	Initial examination	Further examination	Number/Side	Size (cm)	Combined metastasis	Treatment	Outcome^**^ (months)
Nakagawa et al. (7)	57/M	48	Angiography	Angiography/CT/US/Galium-citrate scintigram	1/L	1.0	Brain and lung	Intravascular embolization + Metastasectomy + Interferon	No progression (N/A)
Gal et al. (8)	49/M	Premonitory sign	Symptoms	CT/MRI	1/R	4.0	Adrenal gland	Metastasectomy	Metastasis (25)
Yiotakis et al. (9)	60/M	2	Physical examinations	CT	1/L	1.5	N	Metastasectomy +Interleukin-2 + Interferon	No progression (N/A)
Kang et al. (10)	71/M	144	Physical examinations	MRI/PETCT	1/R	4.1	N	N/A	N/A (N/A)
Landström et al. (11)	59/M	N/A	N/A	CT	1/R	4.3	N/A	Electrochemotherapy	Death (4)
Present case	74/M	58	Physical examinations	US/CT	1/R	1.7	N	Metastasectomy	No progression (1)

^*^The interval from the discovery of RCC to the discovery of muscle metastasis.

^**^Observation time was analyzed in cases without progression. PFS was analyzed in cases with progression. Progression consisted of the state of recurrence, metastasis and death.

RCC, renal cell carcinoma; Ref., reference; M, male; CT, computed tomography; US, ultrasonography; MRI, magnetic resonance imaging; PETCT, positron emission tomography/computed tomography; L, left; R, right; N, no combined metastasis; N/A, not available; cm, centimeters; PFS, Progression-free survival.

Masseter muscles are rare but are possible sites of metastatic RCC. Similar to previous bodies of literature (6), we find that muscle metastases of RCC are more common in lower limbs and trunk, while upper limbs, head and neck are less reported. Compared with thyroid gland, parotid glands, and paranasal sinus, masseter muscles are less common sites involved by RCC at head and neck (9, 12). The rarity of masseter muscle metastases of RCC can be explained such that skeletal structures limit the development of metastatic lesions, which can only develop in natural cavities and loose glands. Besides, the high resistance of muscle to cancer is another reason for the rarity. The resistance may be explained by high tissue pressure, rapid blood flow, lactic acid production, antitumor activity of lymphocytes and natural killer cells, inhibition from skeletal muscle derived peptic factors and protection inhibitors (13, 14). Nevertheless, the special location and surrounding structures of masseter muscles also determine special metastatic pathways. It is considered that, in addition to conventional arterial pathway and lymphatic pathway, vertebral venous plexus may be a special pathway of metastases at head and neck (15). Vertebral venous plexus connects with inferior vena cava and mesenteric vein. Malignant tumors from abdomen and pelvis, like RCC, can be transferred to masseter muscles through this way. The round-trip blood flow and the absence of venous valves also help malignant cells spread in this way. Comprehensive surveillance for patients with RCC is needed, including unusual sites like masseter muscles.

Males seem to be more prone to muscle metastases of RCC, and 88.06% of reviewed patients are males. In particular, all of masseter muscle metastases of RCC are reported in males. This is associated with the male-to-female ratio of about 2:1 in patients with RCC (2). But male predominance is greater in patients with muscle metastases, suggesting that differences in sex hormones may play a role in muscle metastases of RCC. Muscle metastases mainly exist in the elderly, but a few young patients may be related to special types such as Xp11.2 translocation renal cell carcinoma (16) and renal medullary carcinoma (17).

Muscle metastases can be the premonitory or synchronous sign of RCC, but 70.15% of muscle metastases are found after RCC, with a median delay of 60 months. Usually, metastases of RCC occur within 5 years (18), but muscle metastases seem to have a longer delay. Angelini et al. reported a rare muscle metastasis of RCC found more than 40 years later (18). Early dissemination and dormancy are considered to play a role in the delayed metastases of RCC (19). After early dissemination, RCC cells are dormant due to local inappropriate environment and awaken under the stimulation of some factors. Muscles are usually not suitable environment for tumor cells, just as what have been discussed, so it may take more time to wait for awakening. So surveillance for patients with RCC is needed for a long time.

Muscle metastases of RCC tend to be single. All metastatic lesions of RCC in masseter muscles are single. The diameter of muscle metastases is limited to a median of 4.05 cm, because they are usually superficial and easy to be detected. In masseter muscles, limited space may be another reason for limited diameter, with a median of 2.90 cm.

The initial detection and further identification of muscle metastases from RCC are important in diagnosis. Most masseter muscle metastases of RCC are usually superficial and easy to be detected with symptoms or physical examinations such as swelling. Only one masseter muscle metastasis was incidentally found in carotid angiography (7). Muscle masses include a large variety of benign and malignant soft tissue lesions of diverse histological nature (20). In further identification, US is often used as the first imaging examination, for its convenience, free of radiation, and ability to detect blood supply of lesions. In 2019, the characteristics of regular shape and clear boundary in US were not consistent with malignant masses. Benign masses such as hemangioma, lipoma, fibroma and neurogenic tumor, are usually more common than primary and metastatic malignant masses in masseter muscles (21). So a benign mass was considered. Then with clear boundary, high vascularity and absent of neurological symptoms, fibroma was chosen in benign masses as the preliminary consideration. Increased diameter and unclear boundary in CT showed malignant characteristics in 2021. So the mass was removed and pathology finally identified the nature and source of it. In the specimen of present case, Pax-8 was positive, which is related to RCC. The clear cells under microscope, with positive of CA IX, further supported the diagnosis of ccRCC. A lesion from the misdiagnosis can be drawn: 1) the metastatic mass may be regular and well-defined in the early stage, which is easy to disguise as benign mass; 2) the history of malignancy should be considered, though the interval is long; 3) single examination is limited, and multiple examinations are needed for sufficient characteristics of the mass; 4) conclusion from pathology is essential for accurate diagnosis.

For metastases of RCC excepting brain and bone metastases, local complete metastasectomy, systemic immunotherapy and targeted therapy are recommended (22). Complete metastasectomy can lead to better survival and symptom control than incomplete or no metastasectomy (23, 24). According to our review, the most common treatment for muscle metastases is complete metastasectomy. However, structures in masseter area are essential in maintaining facial functions and appearance. In present case, measures were taken to preserve normal function and facial appearance when removing metastases: 1) trying to preserve the masseter muscle on the premise of negative margin; 2) dissecting and preserving the branches of facial nerve; 3) transferring sternocleidomastoid flap to restore facial appearance; and 4) preventing Frey Syndrome with patch and transferred muscle flap. The first two measures had been mentioned to protect masseter muscles and facial nerve (9, 11). We emphasized the restoration of facial appearance and maintaining normal function of sweat glands for the first time with the last two measures. The present patient recovered after complete metastasectomy without malfunction of masseter muscles and other facial structures. Immunotherapy and targeted therapy are often used as adjuvant treatment of surgery or alternative treatment when complete metastasectomy is not suitable. Interferon and interleukin are the main immunotherapeutic agents initially, but they are gradually replaced because of poor effectiveness and tolerance (25). Recently, immune checkpoint inhibitors is the standard form of immunotherapy (26). Among the 28 patients who received any treatment in our review, 14 patients did not have progression with a median observation time of 13.63 months, while 14 patients had final progression with a median progression-free survival (PFS) of 6 months. Most of patients with progression are troubled by combined metastases, leading to worse survival.

We reported a rare case of masseter muscle metastasis of RCC, and collected the clinical characteristics from reviewed literatures of muscle metastases, to provide reference for the diagnosis and treatment. The article is prepared and revised according to the CARE checklist (**Supplementary Table 2**). There are still some limitations in this report. Clinical and molecular characteristics of the primary tumor are insufficient, which may cause deviation in the judgment of metastatic mechanism and process. Because of limited reports of masseter muscle metastases, more cases are needed to collect and summarize their characteristics. Most of the cases reviewed in this paper come from case reports, which are difficult to share a unified standard, leading to several limitations of our conclusions. Further observational and comparative studies are still needed.

## Conclusion

Masseter muscle can be a metastatic site of RCC, and vertebral venous plexus is a possible pathway. Comprehensive surveillance is needed for a long time. Muscle metastases can disguise as a benign mass, while multiple imaging and pathology are important in identifying their sources. If possible, complete metastasectomy with function retention is recommended for muscle metastases.

## Data Availability Statement

The raw data supporting the conclusions of this article will be made available by the authors, without undue reservation.

## Ethics Statement

The studies involving human participants were reviewed and approved by the ethics committee of the Affiliated Hospital of Qingdao University. The patients/participants provided their written informed consent to participate in this study.

## Author Contributions

SW was the patient’s surgeon. FQ and JZ reviewed the literature and contributed to manuscript drafting. XZ and SL analyzed statistical data and contributed to manuscript drafting. ZW and TZ interpreted the imaging and pathological findings. FX, MZ and WJ were responsible for the revision of the manuscript for important intellectual content. All authors listed have made a substantial, direct, and intellectual contribution to the work and approved it for publication.

## Funding

This research was funded by the Science and Technology Program in Chinese Medicine of Shandong Province (No. 2020M074).

## Conflict of Interest

The authors declare that the research was conducted in the absence of any commercial or financial relationships that could be construed as a potential conflict of interest.

## Publisher’s Note

All claims expressed in this article are solely those of the authors and do not necessarily represent those of their affiliated organizations, or those of the publisher, the editors and the reviewers. Any product that may be evaluated in this article, or claim that may be made by its manufacturer, is not guaranteed or endorsed by the publisher.
