# Supplementary material for: Masseter Muscle Metastasis of Renal Cell Carcinoma: A Case Report and Literature Review
Source: Front Oncol. 2022 Feb 2;12:830195. doi: 10.3389/fonc.2022.830195 (PMC8848329; doi:10.3389/fonc.2022.830195)
Supplement: Supplementary file 1 [file Image_1.pdf]

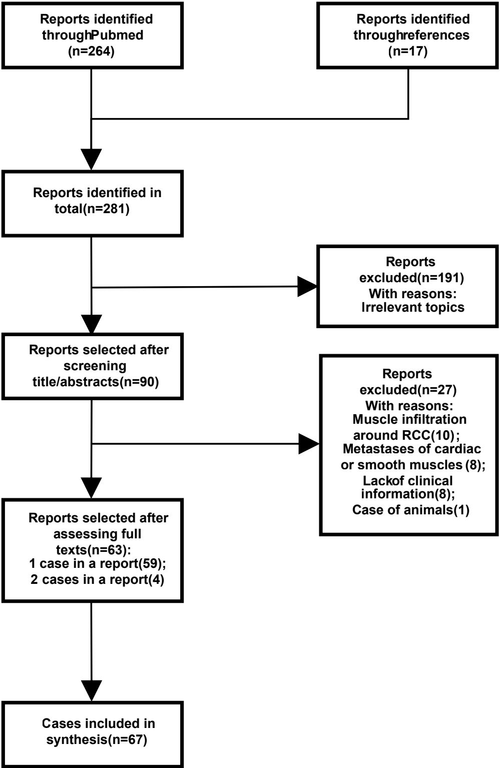
Reports identified throughPubmed (n=264)
Reports identified throughreferences (n=17)
Reports identified in total(n=281)
Reports excluded(n=191) With reasons: Irrelevant topics
Reports selected after screening title/abstracts(n=90)
Reports excluded(n=27) With reasons: Muscle infiltration around RCC(10); Metastases of cardiac or smooth muscles (8); Lackof clinical information(8); Case of animals(1)
Reports selected after assessing full texts(n=63): 1 case in a report(59); 2 cases in a report(4)
Cases included in synthesis(n=67)
