# Supplementary material for: Masseter Muscle Metastasis of Renal Cell Carcinoma: A Case Report and Literature Review
Source: Front Oncol. 2022 Feb 2;12:830195. doi: 10.3389/fonc.2022.830195 (PMC8848329; doi:10.3389/fonc.2022.830195)
Supplement: Supplementary file 2 [file Table_1.docx]

**Table 1 Clinical characteristics of cases with muscle metastasis of RCC**

| **Characteristic** | **Number (%)** | **Mean**±**SD or Median[Quartile]** |
| --- | --- | --- |
| **Age(years)** | 67(100) | 60.31±13.83 |
| **Gender** |  |  |
| male | 59(88.06) |  |
| female | 8(11.94) |  |
| **Interval(months)**^*^ |  |  |
| premonitory sign of RCC | 10(14.93) |  |
| synchronous sign of RCC | 4(5.97) |  |
| delayed sign of RCC | 47(70.15) | 60.00[24.00,144.00] |
| N/A | 6(8.95) |  |
| **Number of MM** |  |  |
| 1 | 51(76.12) |  |
| 2 | 6(8.96) |  |
| 3 | 2(2.98) |  |
| >3 | 4(5.97) |  |
| N/A | 4(5.97) |  |
| **Site of MM**^**^ | 69 |  |
| head and neck | 14(20.29) |  |
| trunk | 18(26.09) |  |
| upper extremities | 12(17.39) |  |
| lower extremities | 25(36.23) |  |
| **Size of MM(cm)**^***^ | 48 | 4.05[2.05,6.50] |
| **Combined metastasis** |  |  |
| Y | 30(44.78) |  |
| N | 35(52.24) |  |
| N/A | 2(2.98) |  |
| **Initial examination** |  |  |
| symptoms | 26(38.81) |  |
| physical examinations | 15(22.39) |  |
| imaging examinations^****^ | 14(20.90) |  |
| N/A | 12(17.90) |  |
| **Further examination** |  |  |
| CT | 31(46.27) |  |
| MRI | 26(38.81) |  |
| US | 14(20.90) |  |
| radionuclide imaging | 14(20.90) |  |
| angiography | 6(8.96) |  |
| N/A | 8(11.94) |  |
| **Treatment** |  |  |
| metastasectomy | 33(49.25) |  |
| immunotherapy | 20(29.85) |  |
| targeted therapy | 10(14.93) |  |
| radiotherapy | 9(13.43) |  |
| chemotherapy | 6(8.96) |  |
| radiofrequency ablation | 1(1.49) |  |
| intravascular embolization | 1(1.49) |  |
| N/A | 14(20.90) |  |
| **Outcome(months)**^*****^ |  |  |
| Without progression | 14(20.90) | 13.63[8.50,42.75] |
| With progression | 14(20.90) | 6.00[4.00,15.00] |
| N/A | 39(58.20) |  |

^*^ The interval from the discovery of RCC to the discovery of muscle metastasis.

^**^ Cases, in which MM were no more than 3, were included. And there were 69 metastatic sites in total.

^***^ Cases, in which MM were no more than 3 and size of MM was available, were included. And there were 48 metastatic sites in total.

^****^ Imaging examinations consisted of CT, MRI, US, radionuclide imaging and angiography.

^*****^ Observation time was analyzed in cases without progression. PFS was analyzed in cases with progression. Progression consisted of the state of recurrence, metastasis and death.

(RCC=renal cell carcinoma; MM=muscle metastases; CT=computed tomography; MRI=magnetic resonance imaging; US=ultrasound; Y=yes; N=no; N/A=not available; SD=standard deviation; cm=centimeters; PFS= Progression-free survival)
