# Supplementary material for: Masseter Muscle Metastasis of Renal Cell Carcinoma: A Case Report and Literature Review
Source: Front Oncol. 2022 Feb 2;12:830195. doi: 10.3389/fonc.2022.830195 (PMC8848329; doi:10.3389/fonc.2022.830195)
Supplement: Supplementary file 3 [file Table_2.pdf]

CARE
case report guidelines

CARE Checklist of information to include when writing a case report

(cc) BY-NC-ND

Topic	Item	Checklist item description	Reported on Line
**Title**	1	The diagnosis or intervention of primary focus followed by the words "case report"	Title
**Key Words**	2	2 to 5 key words that identify diagnoses or interventions in this case report, including "case report"	Lines 101
**Abstract (no references)**	3a	Introduction: What is unique about this case and what does it add to the scientific literature?	Lines 81-83
	3b	Main symptoms and/or important clinical findings	Lines 85-88
	3c	The main diagnoses, therapeutic interventions, and outcomes	Lines 88-92
	3d	Conclusion—What is the main "take-away" lesson(s) from this case?	Lines 93-99
**Introduction**	4	One or two paragraphs summarizing why this case is unique (**may include references**)	Lines 106-118
**Patient Information**	5a	De-identified patient specific information.	Lines 123
	5b	Primary concerns and symptoms of the patient.	Lines 123-129
	5c	Medical, family, and psycho-social history including relevant genetic information	Lines 176-184
	5d	Relevant past interventions with outcomes	Lines 129-130
**Clinical Findings**	6	Describe significant physical examination (PE) and important clinical findings.	Lines 131-175
**Timeline**	7	Historical and current information from this episode of care organized as a timeline	Lines 123-241
**Diagnostic Assessment**	8a	Diagnostic testing (such as PE, laboratory testing, imaging, surveys).	Lines 131-175
	8b	Diagnostic challenges (such as access to testing, financial, or cultural)	Lines 471-478
	8c	Diagnosis (including other diagnoses considered)	Lines 235-237
	8d	Prognosis (such as staging in oncology) where applicable	Lines 238-241
**Therapeutic Intervention**	9a	Types of therapeutic intervention (such as pharmacologic, surgical, preventive, self-care)	Lines 185-187
	9b	Administration of therapeutic intervention (such as dosage, strength, duration)	Lines 188-190
	9c	Changes in therapeutic intervention (with rationale)	Lines 487-493
**Follow-up and Outcomes**	10a	Clinician and patient-assessed outcomes (if available)	Lines 238-241
	10b	Important follow-up diagnostic and other test results	Lines 238-241
	10c	Intervention adherence and tolerability (How was this assessed?)	Lines 239-241
	10d	Adverse and unanticipated events	not available
**Discussion**	11a	A scientific discussion of the strengths AND limitations associated with this case report	Lines 514-526
	11b	Discussion of the relevant medical literature **with references**.	Table 1
	11c	The scientific rationale for any conclusions (including assessment of possible causes)	Lines 368-511
	11d	The primary "take-away" lessons of this case report (without references) in a one paragraph conclusion	Lines 531-537
**Patient Perspective**	12	The patient should share their perspective in one to two paragraphs on the treatment(s) they received	Lines 239-241
**Informed Consent**	13	Did the patient give informed consent? Please provide if requested	**Yes** ☑ **No** ☐
